# Deletion of the NR4A nuclear receptor NOR1 in hematopoietic stem cells reduces inflammation but not abdominal aortic aneurysm formation

**DOI:** 10.1186/s12872-017-0701-4

**Published:** 2017-10-18

**Authors:** Hua Qing, Karrie L. Jones, Elizabeth B. Heywood, Hong Lu, Alan Daugherty, Dennis Bruemmer

**Affiliations:** 1 Department of Medicine, Division of Cardiology, Pittsburgh Heart, Lung, Blood, and Vascular Medicine Institute, UPMC and University of Pittsburgh School of Medicine, Pittsburgh, PA 15261 USA; 2 Graduate Center for Nutritional Sciences, University of Kentucky, Lexington, KY 40536 USA; 3 Department of Endocrinology, the First Affiliated Hospital of Chongqing Medical University, Chongqing, 400016 China; 4 Saha Cardiovascular Research Center, University of Kentucky, Lexington, KY 40536 USA; 5 Department of Physiology, University of Kentucky, Lexington, KY 40536 USA

**Keywords:** Nuclear receptor, Bone marrow transplantation, Aneurysm, Angiotensin II

## Abstract

**Background:**

The NR4A3 orphan nuclear hormone receptor, NOR1, functions as a constitutively active transcription factor to regulate inflammation, proliferation, and cell survival during pathological vascular remodeling. Inflammatory processes represent key mechanisms leading to abdominal aortic aneurysm (AAA) formation. However, a role of NOR1 in AAA formation has not been investigated previously.

**Methods:**

Inflammatory gene expression was analyzed in bone marrow-derived macrophages isolated from NOR1-deficient mice. Low-density lipoprotein receptor-deficient (LDLr^−/−^) mice were irradiated and reconstituted with hematopoietic stem cells obtained from NOR1−/− or wild-type littermate mice. Animals were infused with angiotensin II and fed a diet enriched in saturated fat to induce AAA formation. Quantification of AAA formation was performed by ultrasound and ex vivo measurements.

**Results:**

Among 184 inflammatory genes that were analyzed, 36 genes were differentially regulated in LPS-treated NOR1-deficient macrophages. Albeit this difference in gene regulation, NOR1-deficiency in hematopoietic stem cells did not affect development of AAA formation in bone marrow-derived stem cell transplanted LDLr-deficient mice.

**Conclusion:**

NOR1 deletion induced differential inflammatory gene transcription in macrophages but did not influence AAA formation in mice.

**Electronic supplementary material:**

The online version of this article (10.1186/s12872-017-0701-4) contains supplementary material, which is available to authorized users.

## Background

Abdominal aortic aneurysms (AAA) affect more than 1.1 million people in the United States and bear an 85% chance of death after rupture [1]. Each year in the United States, more than 16,000 deaths result from rupture of abdominal aortic aneurysms, and 33,000 patients undergo repair of asymptomatic abdominal aortic aneurysms to prevent future rupture [2]. Albeit the high morbidity and mortality associated with aneurysm formation, there are no validated or approved medical therapies for attenuating aneurysm growth and rupture. With increasing recognition of the devastating impact of aneurysmal rupture, ongoing research efforts aim to determine mechanisms of initiation and progression of aneurysms.

Medial infiltration of macrophages and the activation of inflammatory signaling represents the earliest histological change during the initiation of AAA formation [3, 4]. Locally secreted inflammatory signals act on these infiltrating myeloid cells by inducing signal-stimulated transcription factors. Among these, the NR4A subgroup of orphan nuclear receptors represents immediate-early response genes, which are expressed rapidly in response to inflammatory stimulation and control essential cellular functions in all processes of pathological vascular remodeling [5, 6]. The NR4A3 subfamily member NOR1 is highly expressed in human abdominal aortic aneurysm tissues [7] and has been implicated previously in proliferation of myeloid progenitor cells [8, 9], recruitment of monocytes to sites of inflammation [10], transcription of inflammatory gene expression [11, 12], and macrophage apoptosis [13]. Based on this key function of NOR1 in the control of inflammation combined with the well-established contribution of macrophage-induced inflammatory signaling to AAA formation [14, 15], we investigated whether NOR1 deletion affects development of AAA.

## Methods

### Cell culture

Bone marrow-derived macrophages (BMDM) were cultured and differentiated as described [16]. Briefly, bone marrow cells were isolated from femurs of NOR1-deficient mice and their wild-type littermates [17], and differentiated in DMEM medium supplemented with 20% fetal bovine serum and 30% L929 cell-conditioned medium for 10 days. Cells were incubated with 100 ng/ml lipopolysaccharides (LPS) at the indicated time points before harvesting total RNA for gene expression analyses.

### Gene expression analysis

RNA was isolated and reverse transcribed as described [9]. Inflammatory gene expression was profiled using nCounter GX mouse inflammation kit consisting of 184 inflammation-related genes and 6 internal reference genes and analyzed by nSolver software 2.6 (NanoString Technologies) as directed by the manufacturer [18]. The in silico analysis for the NOR1 consensus NBRE (NGFI-B response element) motif within differentially regulated genes was performed using the MatInspector software (Genomatix Inc., Ann Arbor, MI). Real-time RT-PCR was performed with the iCycler and SYBR Green I system (Bio-Rad, Hercules, Calif). Each sample was analyzed in triplicate and normalized to transcription factor IIB (TFIIB) mRNA expression, and data was calculated using the 2-ΔΔCt method [19]. Sequences of the primers for RT-PCR were as follows: NOR1 Forward 5′-GGCCGCAGCTGCACTCAGTC -3, Reverse 5′-GCGGAGGGAAGGTCAGCGTG -3′; TFIIB Forward: 5′-CTCTCCCAAGAGTCACATGTCC-3′, Reverse: 5′-CAATAACTCGGTCCCCTACAAC-3′.

### Animals

Male wild-type littermate (NOR1^+/+^) and NOR1-deficient (NOR1^−/−^) mice on a mixed C57BL/6 J/129Sv background were used as described previously [17]. Bone marrow transplantation was performed by tail vein injection of bone marrow-derived cells isolated from NOR1-deficient or wild-type littermate mice to lethally irradiated male LDLr^−/−^ mice purchased from The Jackson Laboratory (Stock # 002207; Bar Harbor, ME) as described [16]. Mice were fed a saturated fat-enriched diet (Harlan Teklad TD88137) for 5 weeks after bone marrow transplantation. One week after initiation of diet feeding, angiotensin II (1000 ng/kg/min) was infused subcutaneously for 4 weeks using Alzet osmotic minipumps (Alzet Model 2004, Durect Corp, Cupertino, CA, U.S.A.). All studies were performed with the approval of the University of Kentucky Institutional Animal Care and Use Committee.

### Systolic blood pressure measurement

Systolic blood pressure was measured on conscious mice using non-invasive BP-2000 blood pressure system (Visitech Systems, Inc., Apex, NC, U.S.A.) during the last 3 days of angiotensin II infusion as described previously [20]. This noninvasive tail-cuff system records tail blood volume changes that correspond to systolic and diastolic blood pressure [21].

### AAA quantification

AAA formation was quantified by measurement of maximal lumen diameters of suprarenal abdominal aortas. High-frequency ultrasound (Visualsonics) was performed on anesthetized mice to measure suprarenal abdominal aortic diameters and areas prior to and on the final day of angiotensin II infusion. Thereafter, aortas were excised at termination, and AAA size was quantified ex vivo by measuring the aorta dissected from sacrificed mice with ImagePro Plus software as described previously [16].

### Statistics

To compare two groups on a continuous response variable, we used a Student *t* test or Mann–Whitney *U* test as appropriate. One-way ANOVA was used to compare multiple groups, followed by Turkey post hoc analysis. All data are represented as means ± SEM. *P* values <0.05 were considered to be statistically significant.

## Results

### Genetic NOR1 deletion altered inflammatory gene expression

**Fig. 1:**
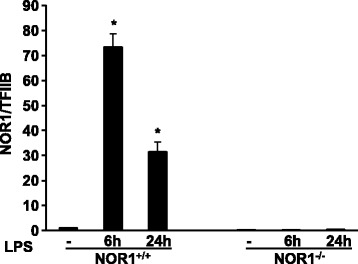
NOR1 expression in wild-type and NOR1-deficient BMDM. BMDM were isolated from NOR1-deficient mice and littermate wild-type control mice. Cells were incubated with 100 ng/ml LPS, and mRNA was harvested at the indicated time points. NOR1 mRNA expression was analyzed by real-time RT-PCR. Data were normalized to transcript abundance of the housekeeping gene TFIIB and presented as fold increase relative to control cells. Values are mean ± SEM (* *P* < 0.05 vs. control cells, *n* = 3 mice/group)

**Fig. 2:**
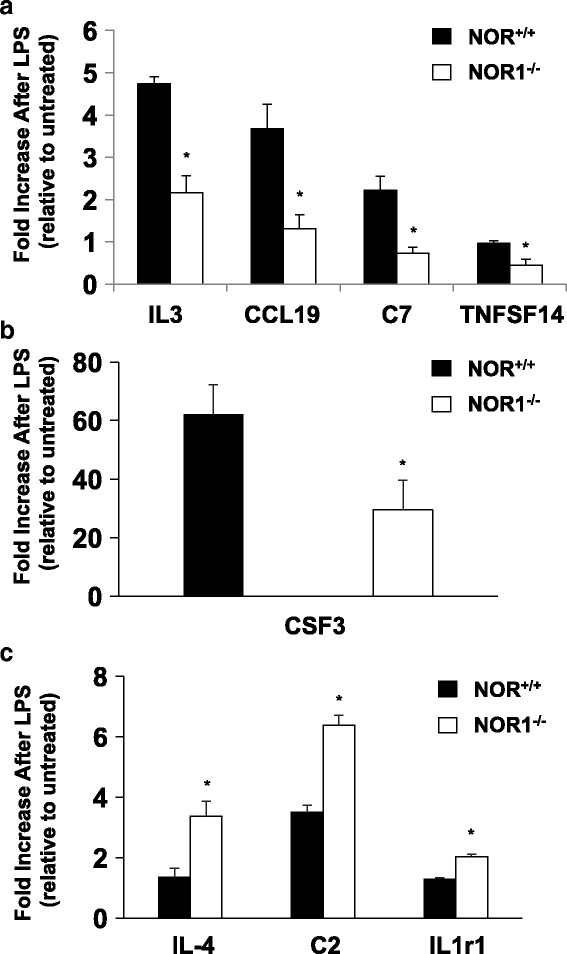
Genetic NOR1 Deletion Impaired Inflammatory Gene Expression. BMDM isolated from NOR1+/+ or NOR1−/− mice (*n* = 6 mice per group) were incubated with 100 ng/ml LPS for 8 h before harvesting RNA for nCounter gene expression profiling. RNA from two mice of the same strain were pooled together and analyzed for transcript expression. **a**-**c**. Histograms of genes which revealed more than 50% difference in transcript abundance in response to LPS-stimulation of NOR1+/+ and NOR1−/− BMDM. Data was processed using the nSolver software and presented as fold increase relative to untreated BMDM of the same genotype. Values are mean ± SEM (* *P* < 0.05 vs. NOR1+/+)

To first investigate the regulation of NOR1 in macrophages, we incubated BMDM isolated from wild-type littermate and NOR1−/− mice with LPS. As depicted in Fig. 1, stimulation of macrophages induced a robust increase in NOR1 mRNA abundance, which was completely absent in cells deficient for the NOR1 locus. Since inducible expression of NOR1 in activated macrophages indicated an important function in control of macrophage inflammation, we next profiled LPS-treated NOR1−/− macrophages for 184 inflammatory gene transcripts using nCounter Gene Expression Assays. Thirty-six genes were differentially regulated in response to LPS treatment of NOR1-deficient macrophages, compared to their wild-type control cells (Additional file 1: Table S1). Among those differentially regulated genes, a large proportion of transcripts were of cytokine and chemokine family of proteins. Figure 2 depicts those eight genes that were at least 50% increased or decreased compared to wildtype cells (Fig. 2a-c). In silico analyses of promoter sequences further identified that 18 genes contained NBRE consensus sequences within their promoters, characterizing these as potential NOR1 target genes (Additional file 2: Table S2).

### Bone marrow deletion of NOR1 did not affect angiotensin II-induced AAA formation

**Fig. 3:**
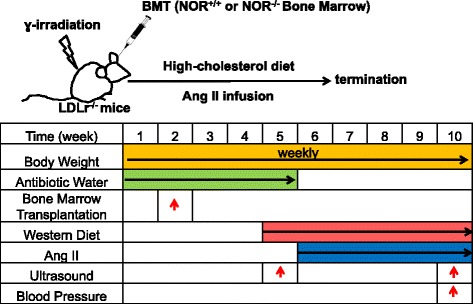
Experimental Design and AAA Formation Study Protocol. LDLr-deficient mice were reconstituted with bone marrow-derived cells from either NOR1+/+ or NOR1−/− mice. Four weeks after engraftment, a diet enriched in saturated fat was fed to transplanted mice beginning one week prior to the start of angiotensin II infusion. The body weight was measured every week; AAA formation was evaluated by ultrasound before and after angiotensin II infusion; blood pressures were measured on three consecutive days before the termination of angiotensin II infusion using a non-invasive blood pressure system

Monocyte recruitment constitutes one of the earliest events during formation of AAA [3, 4]. Because genetic NOR1 deletion altered expression of key inflammatory mediators implicated in monocyte recruitment and their activation, we next investigated whether NOR1 deletion affected AAA formation. One of the widely accepted AAA models includes infusion of angiotensin II into hyperlipidemic mice [20], which induces abdominal aneurysm development with a high degree of rupture and resembles many aspects of human pathology [22]. To generate AAA formation in mice with NOR1 deletion in macrophages, we performed transplantation with bone marrow-derived cells and reconstituted LDLr-deficient mice with either wild-type or NOR1-deficient hematopoietic stem cells. Following reconstitution, mice were fed a saturated fat-enriched diet and infused with angiotensin II using the protocol depicted in Fig. 3.

**Fig. 4:**
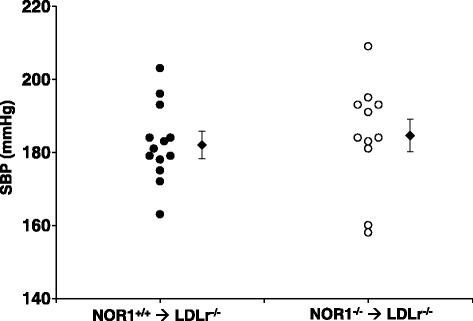
Systolic blood pressure after 28-day angiotensin II infusion in chimeric LDLr^−/−^ mice with macrophage deletion of NOR1. Systolic blood pressure (SBP) was measured on three consecutive days before the termination of angiotensin II infusion using a noninvasive tail-cuff system recording the tail blood volume changes that correspond to systolic and diastolic blood pressure. Filled circles (NOR1^+/+^ donors, *n* = 13) and open circles (NOR1^−/−^ donors, *n* = 11) represent measurements in individual mice; diamonds represent the average for each group, and bars indicate SEM

**Fig. 5:**
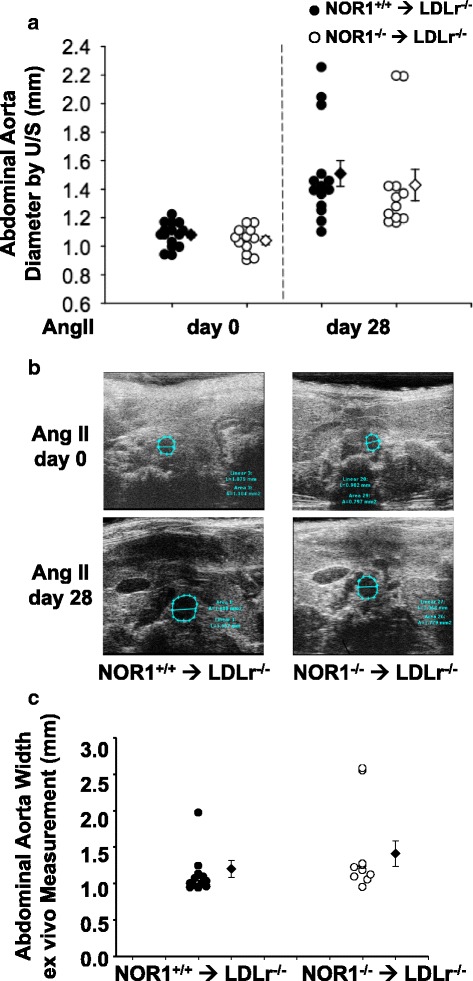
AAA Quantification in chimeric LDLr^−/−^ mice with macrophage-deletion of NOR1. Mice were prepared as indicated in Fig. 3. Recipient LDLr^−/−^ mice transplanted with wild-type or NOR1-deficient bone marrow-derived cells were referred to as NOR^+/+^➔LDLr^−/−^ or NOR1^−/−^ ➔ LDLr^−/−^, respectively **a**. In vivo measurements of suprarenal aorta lumen diameter by ultrasound (U/S) on day 0 and 28 of angiotensin II infusion. *Black circles* (NOR1^+/+^ donors, *n* = 13) and white circles (NOR1^−/−^ donors, *n* = 11) represent measurements in individual mice; diamonds represent the means for each group, and bars indicate SEM. **b**. Representative images of abdominal aorta ultrasound measurements in LDLr^−/−^ mice with wild-type or NOR1-deficient bone marrow transplantation on 0 day and 28 day of angiotensin II (Ang II) infusion. **c**. Ex vivo measurements of the maximal external diameter of suprarenal aorta acquired at termination after 28 days of angiotensin II infusion. *Black circles* (NOR1^+/+^ donors, *n* = 13) and white circles (NOR1^−/−^ donors, *n* = 11) represent measurements in individual mice; diamonds represent the average for each group, and bars indicate SEM

Angiotensin II has well characterized effects to increase blood pressure, although its vascular activity to promote AAA formation are largely independent of this vasopressor function [23]. To assess whether deficiency of the NOR1 locus in hematopoietic cells affected blood pressure, we first performed tail-cuff analyses for blood pressure measurements. As shown in Fig. 4, angiotensin II infusion induced a comparable increase in systolic blood pressure in recipient mice engrafted with either wild-type or NOR1-deficient bone marrow 4 weeks after angiotensin II perfusion (Fig. 4). Suprarenal abdominal aortic lumen diameters and areas were quantified in vivo by ultrasound and ex vivo using morphometric analysis. However, recipient LDLr^−/−^ mice with NOR1 deletion in bone marrow-derived cells showed no difference in AAA size compared to LDLr−/− mice reconstituted with wild-type bone marrow (Fig. 5a-c). Therefore, genetic NOR1 deletion in hematopoietic stem cell has no overt effect on formation of angiotensin II-induced AAA.

## Discussion

NOR1 expression in macrophages regulates proliferation [8, 9], inflammation [11, 12], macrophage lipid-uptake [12], and apoptosis [13]. While these cellular functions have been implicated in initiation of AAA formation [24], it remains currently unknown whether NOR1 contributes to their formation. Therefore, we depleted macrophages of NOR1 using bone marrow transplantation and analyzed whether macrophage NOR1 function affected AAA development in chimeric mice. Although deletion of the NOR1 locus attenuated expression of multiple inflammatory genes, NOR1 deficiency in wall-infiltrating cells derived from hematopoietic progenitors did not affect the initiation of angiotensin II-induced AAA formation.

NOR1 is expressed in all cell types of the vascular wall, including endothelial cells, smooth muscle cells, and infiltrating macrophages [5]. While basal levels of expression are low, NOR1 is induced rapidly in response to mitogenic and inflammatory signaling underlying pathological vascular remodeling [5]. Macrophage activation represents a major mechanism of vascular disease initiation, as inhibition of their infiltration and inflammatory signaling prevents all major vascular diseases, including atherosclerosis and AAA formation [25, 26]. Several prior studies have identified a key role of inducible NOR1 expression in the regulation of macrophage function, including activation of pro-inflammatory [11] but also anti-inflammatory gene regulation [12, 27]. In contrast to these previous studies using viral NOR1 overexpression, our inflammation-biased profiling experiments employed loss-of-function using cells isolated from mice harboring genetic deletion of the NOR1 locus. While different in approach, we observed that 36 out of the 184 analyzed inflammatory genes were differentially expressed, although the magnitude of significant differences was overall mild. Among those regulated genes, the majority were pro-inflammatory, and their expression was decreased in NOR1-deficient cells, including for example IL-6 and TNFα. These loss-of-function studies are consistent with the observations of Pei et al. that NOR1 expression induces transcription of inflammatory genes and activates NF-κB-dependent gene regulation in macrophages [11]. Furthermore, several of the differentially regulated genes revealed putative NBRE binding sites for NOR1, suggesting potentially direct mechanisms of gene regulation. However, in addition to direct gene regulation, NOR1 deletion induces the Ly6-C(hi) monocyte population [8, 9] and is likely to affect myeloid lineage specification, which may further alter inflammatory signaling, particularly in vivo.

Considering the literature supporting an important function of NOR1 in macrophage gene regulation [11, 12, 27], combined with our own observations discussed above, and further that infiltration of circulating monocytes represents one of the earliest mechanisms of AAA formation [4], we tested whether NOR1 deletion in bone marrow-derived cells affected AAA formation and progression. Activation of the renin-angiotensin system constitutes a key mechanism of AAA formation, and infusion of angiotensin II induces AAA formation in hyperlipidemic mice [20], which closely resembles many aspects of human pathology [22]. Unexpectedly, bone marrow cell deletion of NOR1 did not affect formation or rupture incidence of AAA. Since infiltrating macrophages during AAA formation are primarily derived from the bone marrow [28], NOR1 expression in these cells appeared not to be a major determinant of AAA formation, albeit the noted differential gene expression seen in NOR1-deficient macrophages.

There are several potential explanations for these seemingly discrepant observations. First, it is possible that those genes that were regulated by NOR1 are either not causally involved in AAA or that the magnitude of regulation by NOR1 is insufficient to affect the disease development. However, in our in vitro studies, IL-6 expression, for example, was decreased in NOR1-deficient macrophages, and inhibition of IL-6 has been demonstrated to prevent angiotensin II-induced aneurysm formation [29]. Second, it is more likely that there is a balance between the regulation of pro- and anti-inflammatory genes by NOR1, and that the overall net effect on macrophage function does not modulate the initiation and severity of AAA formation. That this hypothesis could be conceivable is supported by the observation that IL-4, which polarizes macrophages towards the more anti-inflammatory M2 phenotype, was increased in NOR1-deficient cells. Third, potential anti-inflammatory effects of NOR1 deletion within locally infiltrating macrophages may become mitigated by an increased number of circulating monocytes. NR4A nuclear receptors have been identified previously as tumor suppressors for myeloid lineage, and deletion of NOR1 increases the Ly6-C(hi) monocyte population [8, 9]. Therefore, NOR1 deficiency specifically in the hematopoietic lineage may increase the number of pro-inflammatory monocytes in the circulation, which could compensate for decreased inflammatory gene expression and ultimately increase AAA formation [28]. Finally, it is possible, if not likely, that decreased inflammation of infiltrating NOR1-deficient macrophages during angiotensin II-induced vascular remodeling may be compensated by an increase in inflammatory gene expression or matrix remodeling enzymes in resident endothelial and vascular smooth muscle cells. Although we have not performed an extensive histological examination of tissues, this notion is supported by the recent findings that expression of NOR1 in endothelial cells increased monocyte adhesion through the regulation of VCAM-1 and ICAM-1 [30], which constitutes an important early mechanism of AAA formation [31]. However, dissecting the more cell-specific contribution of NOR1 in endothelial cells, smooth muscle cells, and mature macrophages will depend on genetic deletion using conditional, cell-specific gene targeting in future experiments.

## Conclusion

In conclusion, our data demonstrate that genetic NOR1 deletion induces moderate effects on macrophage inflammatory gene expression but does not affect angiotensin II-induced AAA formation.

## Additional files

Additional file 1: Table S1. Inflammatory gene expression in response to LPS treatment of bone marrow-derived macrophages using nCounter Gene Expression Assays. (PDF 61 kb)

Additional file 2: Table S2. List of genes that were differentially regulated by NOR1 deletion and contain NGFI-B-responsive element (NBRE) binding sites in the 5′ untranslated region (5’UTR). (PDF 22 kb)

## Abbreviations

AAA: Abdominal aortic aneurysm
BMDM: Bone marrow-derived macrophages
ICAM-1: Intercellular adhesion molecule-1
LDLr: Low density lipoprotein receptor
LPS: Lipopolysaccharide
NOR1: Neuron-derived orphan receptor 1
VCAM-1: Vascular cell adhesion molecule-1
