# Supplementary material for: Deletion of the NR4A nuclear receptor NOR1 in hematopoietic stem cells reduces inflammation but not abdominal aortic aneurysm formation
Source: BMC Cardiovasc Disord. 2017 Oct 18;17:271. doi: 10.1186/s12872-017-0701-4 (PMC5648424; doi:10.1186/s12872-017-0701-4)
Supplement: Supplementary file 1 — Inflammatory gene expression in response to LPS treatment of bone marrow-derived macrophages using nCounter Gene Expression Assays. (PDF 61 kb) [file 12872_2017_701_MOESM1_ESM.pdf]

Supplementary Table 1

Gene Name	mean NOR1$^{+/+}$	mean NOR1$^{-/-}$	SEM NOR1$^{+/+}$	SEM NOR1$^{-/-}$	P
Atf2	0.75602024	0.752796192	0.004990271	0.020296109	0.884877731
Bcl6	1.40644687	1.50677788	0.110574049	0.073976821	0.492722532
C1qa	1.053876417	1.039610265	0.145898863	0.114730957	0.942423992
C1qb	1.305216344	1.194361595	0.089009182	0.059542276	0.359065489
C1r	3.12229738	2.472212766	0.290342981	0.142485071	0.11479542
C1s	2.570592485	1.444742914	0.509679815	0.147537813	0.101134004
C2*	3.495408425	6.386820017	0.237859488	0.327950446	0.002038331
C3	7.844954361	7.534259983	0.581445002	0.365390338	0.674410287
C3ar1	1.361837068	1.231228413	0.03960316	0.029267376	0.05684717
C4a*	5.325106523	3.76178351	0.133590065	0.364891054	0.015822355
C6	2.497287777	1.475304195	0.749246451	0.409128345	0.297339876
C7*	2.218996082	0.731505276	0.331266201	0.146448225	0.0147696
C8a*	3.259487261	1.968128181	0.41806256	0.143051858	0.043133279
C8b	1.330948556	1.609904045	0.13595123	0.078428121	0.150149418
C9	1.622683977	2.382750723	0.280600312	0.441652051	0.219990455
Ccl11	1.182567411	1.303241805	0.478752276	0.334754808	0.846433724
Ccl17	1.256819825	1.149626698	0.284321295	0.336463764	0.81971176
Ccl19*	3.672759192	1.312437917	0.578603382	0.334181397	0.024176393
Ccl2	26.27597562	25.74132088	1.519954714	1.318100552	0.803567273
Ccl21b	1.051424478	0.622499452	0.08398941	0.248860959	0.177794798
Ccl22	1.024219858	1.353660461	0.29342226	0.195427567	0.402967668
Ccl24*	2.202653166	1.536600619	0.140994146	0.119214142	0.022610132
Ccl3*	12.15571645	10.06982304	0.244693052	0.299357413	0.005711422
Ccl4	16.21295974	13.53103184	1.018665254	0.513398993	0.078423666
Ccl5	1223.7018	1188.68043	42.38619105	31.46330779	0.543331856
Ccl7	61.09449467	63.86456686	4.419659834	5.582674292	0.717072478
Ccl8	34.43762421	26.33014127	5.338840752	3.301634956	0.266085974
Ccr1	1.399134919	1.265176769	0.04312115	0.314805672	0.694994435
Ccr2	0.557959631	0.635080528	0.090510837	0.078840266	0.555525854
Ccr3	4.894669178	3.761901171	0.412124846	0.358716788	0.106836606
Ccr4	1.21719864	1.696997613	0.240819015	0.126159444	0.152352177
Ccr7*	4.238661103	2.187498067	0.320864111	0.308474544	0.009967927
Cd4	0.84813218	0.914249653	0.108757441	0.126163309	0.711681815
Cd40*	55.18932265	49.86945233	0.943798775	1.480176771	0.03876338
Cd40lg	0.861688617	1.629871128	0.156142754	0.381895809	0.136103179
Cd55*	0.86356158	0.752296227	0.019491094	0.0032587	0.004895263

Gene Name	mean NOR1$^{+/+}$	mean NOR1$^{-/-}$	SEM NOR1$^{+/+}$	SEM NOR1$^{-/-}$	P
Cdc42	1.1300344	1.075436951	0.017681059	0.02498492	0.149031911
Cebpb	3.057477259	2.874888833	0.167080921	0.157087299	0.470507016
Cfb	10.07907922	9.158800411	0.616693063	0.481921698	0.304867195
Cfd	0.826048162	0.905712984	0.18245277	0.241195353	0.805243357
Cfl1	0.946533922	0.870266606	0.049680152	0.009122613	0.205576188
Cltc	1.001346467	0.95550412	0.007892932	0.023532053	0.138476968
Creb1	0.667563605	0.784686172	0.090679918	0.074566823	0.374921372
Crp	3.555078646	2.062080353	0.785225336	0.557202493	0.195929757
Csf1	28.75754777	28.72814073	0.766366727	2.082882303	0.990062847
Csf2	3.791270907	3.603956468	0.133619439	0.534088937	0.750798766
Csf3*	62.20234493	29.59749824	5.880221848	3.777010879	0.009551552
Cxcl1	21.21969218	16.77520573	2.756627732	1.705595022	0.242243603
Cxcl10	477.1251569	546.026342	35.18469784	15.62264322	0.147989566
Cxcl2*	19.25545545	11.04053347	1.175353685	0.75379881	0.004171786
Cxcl3*	2.860182926	1.826170327	0.088401632	0.197369983	0.008767377
Cxcl5	3.176050182	2.634081178	0.260230381	0.523987219	0.406688752
Cxcl9	528.4479734	613.6034756	93.53346266	74.74627354	0.516222239
Cxcr4	0.049156655	0.065073918	0.001365641	0.01043533	0.204968294
Daxx	9.355024124	9.297825364	0.530461218	0.169538343	0.923136272
Ddit3*	1.19957163	1.035589396	0.029638201	0.011277432	0.00664691
Elk1*	1.630169142	1.161738297	0.121991272	0.073666886	0.030299685
Fasl	2.290535306	1.572526027	0.345698365	0.177585877	0.138394127
Fos	0.929913906	1.060797646	0.044067818	0.092995587	0.272342561
Fxyd2	0.506373715	0.429099903	0.037553871	0.037664501	0.219918756
Gapdh	1.088359581	1.045364056	0.028231433	0.042262864	0.445216214
Gnaq	0.551203319	0.502671044	0.017596157	0.010843475	0.078680571
Gnas	0.856294097	0.813967893	0.023049458	0.015983452	0.205798218
Gnb1*	1.171083729	1.096442154	0.004448484	0.009677233	0.002182621
Gngt1	2.616751586	1.504231327	0.430781767	0.309105655	0.103855209
Grb2	0.963090555	0.920928291	0.026870454	0.017660801	0.259983574
Gusb*	0.55191977	0.510093965	0.011144623	0.009794462	0.047874995
H2-Ea	2.276318035	1.454444387	0.279536941	0.154927177	0.061875341
H2-Eb1	1.753461527	1.01476628	0.277408783	0.211612835	0.101665204
Hc	1.043148444	1.602190573	0.146328093	0.154084636	0.058134276
Hdac4	0.345569415	0.469436431	0.058508627	0.055464622	0.19924015
Hmgn1	0.566416855	0.547655405	0.033622524	0.021357908	0.662173696
Hprt1	0.688009521	0.684197219	0.023366594	0.023943118	0.91476684
Hras1	0.794341735	1.054481315	0.043147417	0.088135274	0.056924413

Gene Name	mean $NOR1^{+/+}$	mean $NOR1^{-/-}$	SEM $NOR1^{+/+}$	SEM $NOR1^{-/-}$	P
Hspb2	3.185327922	2.102946684	0.680676605	0.396608955	0.241424267
Ifna1	1.925450062	1.490472815	0.181078042	0.142353647	0.131984742
Ifnb1	6.073324881	5.547133363	1.162048873	0.253738557	0.681075142
Ifng	0.892884168	0.900028823	0.024249585	0.20452523	0.973989098
Il10	2.305266553	2.311210959	0.291904207	0.245278123	0.988307398
Il10rb	1.122595193	1.079584412	0.048353208	0.032836543	0.502630497
Il11	1.678442503	2.027831369	0.383371979	0.142424503	0.441080268
Il12a*	2.596390958	1.427055788	0.034776053	0.134560997	0.001092442
Il12b	222.6170242	188.0131698	36.72193991	37.80978235	0.547336134
Il13	1.030524433	1.392234974	0.141346293	0.272679077	0.304205509
Il15	9.022684864	10.09817005	0.487781932	0.355774372	0.149447216
Il18	11.26134577	11.35688643	0.688772332	0.876101846	0.935800571
Il18rap	2.119155183	2.883156436	0.165312882	0.652659839	0.319863901
Il1a	84.61801395	52.83385663	11.39285436	5.973758225	0.068889036
Il1b	222.4241182	142.5910207	30.81908928	20.02884682	0.095595854
Il1r1*	1.280431369	2.042523108	0.04736756	0.062030669	0.000616306
Il1rap	2.320855995	2.293527958	0.015537341	0.137557526	0.853131704
Il1rn	41.01765235	41.43671564	5.331106656	4.805529642	0.956240661
Il2	1.423308857	1.324617717	0.256943556	0.43767106	0.855294249
Il22	1.615606496	1.306307426	0.327414392	0.231841959	0.483743453
Il22ra2	1.86444858	1.358773146	0.278337039	0.272027997	0.263671036
Il23a	1.593413755	1.494590609	0.699606894	0.703967571	0.925475042
Il23r*	25.14447995	19.71381512	0.783027841	0.685403232	0.006433482
Il3*	4.739823904	2.167244815	0.169631571	0.393285164	0.003867463
Il4*	1.356966569	3.362544991	0.287032439	0.495255199	0.024813363
Il5	2.170630363	0.745549342	0.713220894	0.067026577	0.117541086
Il6*	140.1146014	82.70517051	7.777720247	6.633369209	0.004940434
Il6ra	0.459831013	0.439126771	0.014922016	0.009226843	0.303341866
Il7	5.029519426	4.706478606	0.407476242	0.522595921	0.651430841
Il8ra	1.452085606	1.141820641	0.149651058	0.073742956	0.136443622
Il8rb	1.149108948	1.409788587	0.232709709	0.13237553	0.385349343
Il9	1.306363392	1.808068737	0.13336443	0.319053918	0.220446242
Itgb2	0.781091989	0.752185161	0.024066374	0.045104288	0.601987481
Jun	0.820540301	0.784037971	0.048675937	0.036530658	0.580971899
Keap1	2.834939892	2.698105729	0.120460642	0.059611644	0.366206897
Kng1	1.441330822	1.923995307	0.155590886	0.747282979	0.561511144
Limk1*	0.565354372	0.666571545	0.018547086	0.011715106	0.009926016
Lta	5.33226709	6.285034286	0.541633105	0.462191303	0.251866684

Gene Name	mean NOR1$^{+/+}$	mean NOR1$^{-/-}$	SEM NOR1$^{+/+}$	SEM NOR1$^{-/-}$	P
Ly96	1.281155189	1.272989392	0.041617228	0.010818586	0.858634668
Maff	6.271689928	7.149109686	0.168744325	0.980842137	0.427780628
Mafg	0.683027562	0.777134728	0.024112564	0.026693593	0.059037792
Mafk	3.144409461	3.124993569	0.158778237	0.088925956	0.920171825
Map2k1	2.924712988	2.798289381	0.110482369	0.07254169	0.392971702
Map2k4	1.701743949	1.569520539	0.053425717	0.09490953	0.291512909
Map2k6	0.565245811	0.700136731	0.053693863	0.018239391	0.076094602
Map3k1	0.519460141	0.56431099	0.016124717	0.014455176	0.107095269
Map3k5	0.793824141	0.860660818	0.047967341	0.036186152	0.328329334
Map3k7	0.835935262	0.804186104	0.00735092	0.012917169	0.099510169
Map3k9	1.439372079	0.955853052	0.258229955	0.059812697	0.142191351
Mapk1	0.78208656	0.804743892	0.007708349	0.007709966	0.106239418
Mapk14	0.379609167	0.345215466	0.018500356	0.005334195	0.148587173
Mapk3	0.677592821	0.66805157	0.015157234	0.020862241	0.730139603
Mapk8	1.125180509	0.959064124	0.052386798	0.036478627	0.059907616
Mapkapk2*	3.471743018	3.264332789	0.030739339	0.036381993	0.012112618
Mapkapk5	1.148480179	1.046103745	0.049648005	0.011377036	0.114805794
Masp1	1.130445178	1.210199706	0.081021086	0.153696155	0.670045626
Masp2	0.636334345	1.109378467	0.147018663	0.115189639	0.064475181
Max*	2.324903187	2.117854763	0.035838309	0.052909319	0.031674918
Mbl2	1.509580463	1.13286674	0.042215759	0.286492396	0.263181413
Mef2a	0.471854096	0.488763747	0.029427898	0.029644827	0.706325128
Mef2b	1.452748983	1.094164239	0.227300747	0.32848325	0.420105128
Mef2c	0.256255104	0.282842969	0.021351688	0.008489323	0.311614035
Mef2d	0.65663222	0.613973822	0.026900273	0.018497745	0.261384313
Mknk1	0.700939183	0.748362675	0.011490629	0.015152481	0.067211284
Myc	1.002085	0.864174662	0.080747919	0.040465652	0.20149271
Myd88	3.313269863	3.130998816	0.074525241	0.018938264	0.076785531
Myl2	1.008197338	1.68176359	0.062836748	0.293833972	0.088459817
Nfatc3	0.746585992	0.732099175	0.025925283	0.008146137	0.622210437
Nfe2l2	1.419778971	1.321493713	0.021539726	0.049364336	0.142072768
Nfkb1*	2.179133807	1.978733644	0.036817553	0.040030636	0.021116973
Nos2	72.09795347	71.33800211	9.005173978	5.68865247	0.946546383
Nox1*	1.299651733	1.137092179	0.042124672	0.016806611	0.023079529
Nr3c1	1.273787081	1.193879164	0.013260471	0.03636942	0.10793796
Pdgfa	0.911396215	0.989072741	0.039600387	0.123486258	0.581455927
Pgk1	1.365962287	1.385405122	0.024803471	0.062179425	0.785919167
Pik3c2g	0.980409056	0.766699854	0.074596455	0.137458056	0.243564227

Gene Name	mean NOR1$^{+/+}$	mean NOR1$^{-/-}$	SEM NOR1$^{+/+}$	SEM NOR1$^{-/-}$	P
Pla2g4a	2.631420005	2.676463728	0.023857455	0.137843509	0.763587394
Plcb1	0.280936277	0.263425025	0.053328206	0.026125473	0.782755232
Ppp1r12b	0.593763836	0.755605694	0.15570481	0.19583585	0.552962007
Prkca	0.792389812	1.510122146	0.126390767	0.281434304	0.080566678
Prkcb1	0.639904376	0.584179894	0.035928404	0.013048993	0.218633781
Ptk2	2.151789944	2.072930906	0.051001886	0.025109114	0.237670213
Rac1	0.95637635	0.923926357	0.010055345	0.021818383	0.248135946
Raf1	0.964851184	0.934133851	0.02942749	0.02861551	0.495849348
Rapgef2	9.906402855	9.386204413	0.345858006	0.133171892	0.233099837
Rhoa	0.928446517	0.887409273	0.015325211	0.014826079	0.126607635
Ripk1	0.81466039	0.802597214	0.017620653	0.011530066	0.597402669
Ripk2	1.930913683	1.971753132	0.060316307	0.037273846	0.59548314
Rock2	0.702276127	0.648468892	0.028403054	0.016229283	0.175347925
Rps6ka5*	0.213878285	0.282680889	0.007045548	0.010106983	0.005042578
Shc1	0.8208192	0.847203697	0.037592067	0.015863516	0.553095149
Stat1*	10.97652536	12.45494401	0.158426077	0.317261049	0.014041919
Tgfb1*	0.790631351	0.719324356	0.003391805	0.010142069	0.002628734
Tgfb2	3.125758118	3.747699256	0.75402245	0.209318083	0.471236923
Tgfb3	1.700132506	2.318151463	0.035553867	0.270190579	0.085936732
Tgfbr1	0.446183114	0.421867538	0.016681927	0.029247401	0.510155679
Tlr1*	1.744959469	1.483711552	0.056053887	0.067009509	0.040323426
Tlr2*	0.807556485	0.725945301	0.012241502	0.020459552	0.026707009
Tlr3	14.67023452	14.35064233	0.810326666	0.445386759	0.747031762
Tlr4	1.119947745	1.158990306	0.124919787	0.07472527	0.801796948
Tlr5	0.895186336	0.874378836	0.336213146	0.308986497	0.96583921
Tlr6	1.587591458	1.630175127	0.053651515	0.066457862	0.644251763
Tlr7	2.292058445	2.155721109	0.084339616	0.046989982	0.23076428
Tnf*	8.355466146	7.492937558	0.073305398	0.069586952	0.00103484
Tnfsf14*	0.965524914	0.448583387	0.063161513	0.14273086	0.029599162
Tollip	1.181558303	1.152243634	0.0359171	0.035032366	0.590400501
Tradd*	1.127688984	1.014333446	0.030403255	0.017446522	0.031860597
Traf2	2.000929276	1.969779927	0.047364927	0.035146844	0.62535317
Tubb5	0.635728393	0.639279325	0.013289209	0.01832079	0.8829309

Supplementary Table 1. Inflammatory gene expression in response to LPS treatment of bone marrow-derived macrophages using nCounter Gene Expression Assays. Macrophages were isolated from NOR1-deficient or wild-type mice (n=6 mice per group) and treated with 100 ng/ml LPS for 8 hours. RNA from two mice of the same strain was pooled and analyzed for transcript expression. After standard normalization to six house-

keeping genes, the LPS response was expressed as fold increase over basal transcript levels. Data are represented as mean and standard error of the mean (SEM). Significantly different gene expression between NOR1+/+ and NOR1-/- cells is indicated in yellow ($P<0.05$).
