# Supplementary material for: Deletion of the NR4A nuclear receptor NOR1 in hematopoietic stem cells reduces inflammation but not abdominal aortic aneurysm formation
Source: BMC Cardiovasc Disord. 2017 Oct 18;17:271. doi: 10.1186/s12872-017-0701-4 (PMC5648424; doi:10.1186/s12872-017-0701-4)
Supplement: Supplementary file 2 — List of genes that were differentially regulated by NOR1 deletion and contain NGFI-B-responsive element (NBRE) binding sites in the 5′ untranslated region (5’UTR). (PDF 22 kb) [file 12872_2017_701_MOESM2_ESM.pdf]

Gene	Sequence
C2	-2520 -2506<br>5’-acgAAAGGGCAatgc-3’
C4a	-1973 -1959<br>5’-gaacAAGGTCActga-3’
C8a	-1323 -1309<br>5’-gagAAAGGTGAggtc-3’
CCR7	-2228 -2214<br>5’-tggAAAGGACAtggg-3’
CD40	-1964 -1950<br>5’-gcttAAGGTCAatac-3’
CSF3	-705 -691<br>5’-aggcAAGGTCAggtg-3’
Elk1	-1693 -1679<br>5’-aaaAAAGGGCAaaat-3’
Gnb1	-629 -615<br>5’-tggAAAGGTCTcaga-3’
IL23r	-232 -218<br>5’-gtaAAAGGTCTcaat-3’
Limk1	-2682 -2668<br>5’-tctgAAGGTCAgaag-3’
Mapkapk2	-2682 -2668<br>5’-tccAAAGGACAaggc-3’
Max	-892 -878<br>5’-gctAGAGGTCAaacc-3’
NFkb1	-682 -668<br>5’-ctttAAGGTCActta-3’
Nox1	-2577 -2563<br>5’-ggaAAAGGTTAatgg-3’
Rps6ka5	-1635 -1621<br>5’-attAAAGGTCAgtgc-3’
STAT1	-2321 -2307<br>5’-aaggAAGGTCAtgct-3’
Tgfb1	-1056 -1042<br>5’-agaAAAGGACAaagg-3’
Tlr1	-675 -661<br>5’-gacAAAGGTGAcaga-3’

Supplementary Table 2. List of genes that were differentially regulated by NOR1 deletion and contain NGFI-B-responsive element (NBRE) binding sites in the 5’ untranslated region (5’UTR). The NBRE consensus sequence consists of an octanucleotide AAAGGTCA motif and is labeled in capital letters in the Table. The numbers above the DNA sequences indicate the start and end position upstream of the translation start codons.
